# The telomere lengthening conundrum—artifact or biology?

**DOI:** 10.1093/nar/gkt370

**Published:** 2013-05-11

**Authors:** Troels Steenstrup, Jacob v. B. Hjelmborg, Jeremy D. Kark, Kaare Christensen, Abraham Aviv

**Affiliations:** ^1^Department of Biostatistics, Institute of Public Health, University of Southern Denmark, Odense, Denmark, ^2^The Danish Aging Research Center, University of Southern Denmark, Odense, Denmark, ^3^Department of Clinical Biochemistry and Pharmacology, Odense University Hospital, Odense, Denmark, ^4^Department of Clinical Genetics, Odense University Hospital, Odense, Denmark, ^5^The Hebrew University–Hadassah School of Public Health and Community Medicine, Ein Kerem, Jerusalem, Israel and ^6^The Center of Human Development and Aging, University of Medicine and Dentistry of New Jersey, New Jersey Medical School, Newark, NJ, USA

## Abstract

Recent longitudinal studies of age-dependent leukocyte telomere length (LTL) attrition have reported that variable proportions of individuals experience LTL lengthening. Often, LTL lengthening has been taken at face value, and authors have speculated about the biological causation of this finding. Based on empirical data and theoretical considerations, we show that regardless of the method used to measure telomere length (Southern blot or quantitative polymerase chain reaction-based methods), measurement error of telomere length and duration of follow-up explain almost entirely the absence of age-dependent LTL attrition in longitudinal studies. We find that LTL lengthening is far less frequent in studies with long follow-up periods and those that used a high-precision Southern blot method (as compared with quantitative polymerase chain reaction determination, which is associated with larger laboratory error). We conclude that the LTL lengthening observed in longitudinal studies is predominantly, if not entirely, an artifact of measurement error, which is exacerbated by short follow-up periods. We offer specific suggestions for design of longitudinal studies of LTL attrition to diminish this artifact.

## INTRODUCTION

Telomere length (TL) on average declines with age. However, longitudinal studies in humans report varying proportions of subjects who ostensibly display TL lengthening over time (1–12). In this communication, we address the following question: does the lengthening of TL represent a true biological phenomenon or an artifact inherent in the measurement error of TL in relation to the duration of the follow-up period? Biological ‘noise’, arising from neither genes nor the environment, is a major explanation for the inter-individual variation in phenotypic expressions (13). This form of noise is largely beyond our control. However, the impact of noise due to measurement error on research findings can be minimized both by optimizing assays and improving study design.

As most epidemiological telomere research has focused on leukocyte TL (LTL) dynamics (LTL and its age-dependent attrition), we examined this key question with respect to LTL. If age-dependent LTL attrition, which ultimately reflects TL shortening in hematopoietic stem cells (14), is indeed halted or reversed at any time of the human life course, major efforts should be invested to explain the etiology of this phenomenon. Reversal of LTL attrition could originate from altered telomere dynamics at any level of the hematopoietic hierarchy, including hematopoietic stem cells at the top and extending down through hematopoietic progenitor cells to circulating leukocytes at the bottom. However, if LTL elongation is an artifact, researchers need to optimize longitudinal studies of LTL dynamics (by using both measurement methods that minimize error and performing studies with extended follow-up) so that the overall effect of this artifact will be minimized or eliminated.

To this end, based on empirical data available from published longitudinal studies, the duration of which has ranged from 6 months to 13.1 years (Table 1), we performed a series of analyses with a view to further explore the relation between change in LTL over time, LTL measurement error and the length of the follow-up. In discussing our results, we seek potential explanations for discrepancies between the published empirical findings and the outcomes of these computations. We further propose a way forward with respect to the measurement of LTL and study design in evaluating human telomere dynamics.

**Table 1.** Empirical and computational data related to longitudinal studies of LTL attrition

Study	Year	(*N*)	FU (years)	LTLb (kb; T/S)	LTLfu (kb; T/S)	Change (bp/yr; T/S)	Method	CV (%)	Rep	Observed G (%)	Predicted G (%)
Gardner *et al.* (1)^a^	2005	70	11.5	7.58	7.22	31.3	SB	1.5	2	7.9 (2.8–16.8)	0.1
Martin-Ruiz *et al.* (3)^b^	2005	67	3.7				qPCR			^c^	
Martin-Ruiz *et al.* (3)^b^	2005	14	12.9				qPCR			^c^	
Aviv *et al.* (2)^a^	2009	635	5.9	7.45	7.23	40.7	SB	1.4	2	11.2 (8.8–13.9)	1.0
Ehrlenbach *et al.* (4)	2009	510	10	8.02^d^	7.44^d^	45.5^d^; 0.034	qPCR	0.9	1	15.9 (12.8–19.3)	0.0
Nordfjäll *et al.* (5)	2009	959	10				qPCR	6.0		34 (31–37)^e^	
Epel *et al.* (6)	2009	134	2.5	4.70^d^	4.70^d^	^f^	qPCR	7.0		47 (38–56)	
Farzaneh-Far *et al.* (7)	2010	608	5	5.50^d^	5.29^d^	42^d^	qPCR	3.7	1	39 (35–43)	22.8
Chen *et al.* (8)^a^^,^^b^	2011	271	5.8	7.37	7.18	31.4	SB	2.4	2	14.4 (10.4–19.1)	14.8
Chen *et al.* (8)^a^^,^^b^	2011	271	6.6	7.18	6.94	33.5	SB	2.4	2	10.7 (7.3–15.0)	9.6
Chen *et al.* (8)^a^^,^^b^	2011	271	12.4	7.37	6.94	32.2	SB	2.4	2	1.5 (0.4–3.7)	1.0
Svenson *et al.* (9)	2011	50	0.5	0.578	0.577	0.001	qPCR	6.0	1	50 (36–64)	50.0
Kark *et al.* (10)	2012	609	13.1	7.33	7.00	25.2	SB	2.2	2	3.0 (1.8–4.7)	1.8
Shalev *et al.* (11)^g^	2012	236	5	1.08	0.96	0.024	qPCR			16.9 (12.4–22.4)^h^	
Steenstrup *et al.* (12)	2013	80	10.9	5.84	5.51	30.8	SB	2.8	2	7.5 (2.8–15.6)	1.7

Year = year of publication; *N* = sample size; FU = follow-up duration; LTL is expressed in bp for the SB, T/S units for the qPCR method or both, if the transformed T/S units are also expressed in absolute LTL; LTLb = LTL at baseline; LTLfu = LTL at follow-up; CV = inter-assay coefficient of variation; Rep = inter-assay replicate number (1 is used when this is not provided); Observed G = observed percentage of LTL gainers with 95% confidence interval (in brackets); Predicted G = the predicted percentage of LTL gainers during the follow-up.

^a^Various subsets of the Bogalusa Heart Study, described in different publications.

^b^Same study, but LTL change was measured at different follow-up intervals.

^c^Overall, no change in LTL during follow-up, but percentage of LTL not gainers reported.

^d^T/S data converted to absolute LTL units (bp).

^e^Study provided the value of ‘stable or increased’ LTL jointly. Half of the individuals with ‘stable’ LTL could not be computed, based on the principle applied to the other studies. Thus, this value might be an overestimate of LTL gainers.

^f^Baseline and follow-up LTL and T/S was reported to be identical (up to the precision used in the article), thus the change cannot be computed.

^g^Study consisted of children aged 5–10 years old.

^h^Only the value of LTL gainers (>15% lengthening) was reported; hence, this value might be an underestimate of LTL gainers.

Blank entries denote parameters not reflect unreported data or not computed owing to insufficient information.

The percentage of LTL gainers could not be predicted in several studies for the following specific reasons: for (6), the mean change between baseline and follow-up LTL was zero within the precision used to report values; (11) reported intra-assay CV for T and S separately, but not inter-assay for T/S ratio. For (4), the unusually low 0.9% CV is assumed to be the inter-assay CV. For (3) and (5), no data were provided about LTLb and LTLfu to compute Change.

## MATERIALS AND METHODS

Measurement error can lead to observations that LTL spuriously seems to become longer over time in a subset of individuals even under an assumption that LTL undergoes age-dependent attrition in all individuals. This can be quantified by denoting μ as the true LTL attrition over a fixed period and assuming that it is the same for all individuals. This assumption does not typically hold in epidemiological and biological settings, but we apply it here to enable use of the limited information available from published papers. A random effects model, which includes not only the mean LTL attrition but also a term that can capture individual deviations from the mean attrition, would give a more precise estimate. However, such a model would require the entire data set of a given study.

Because of measurement error, observations of an individual’s change in LTL will deviate from μ. We assume that the measurement error of LTL is normally distributed around the true value of LTL. We denote the standard deviation (SD) by σ and assume that σ is the same for all individuals. As measurement error occurs for baseline and follow-up samples, the σ of the difference between baseline and follow-up (σ_d_) will be larger than σ. From the properties of the normal distribution, it follows that 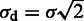. This increase in measurement error can be compensated for by performing replicates of baseline and follow-up. If, for instance, duplicate measurements of both baseline and follow-up are performed (i.e. a total of four measurements for each individual), then the mean of the two differences will give a more precise estimate of the true difference than each of the differences on their own. In this case, σ_d_ = σ. Further increasing the number of replicates will increase precision and thus decrease σ_d_.

**Figure 1.**
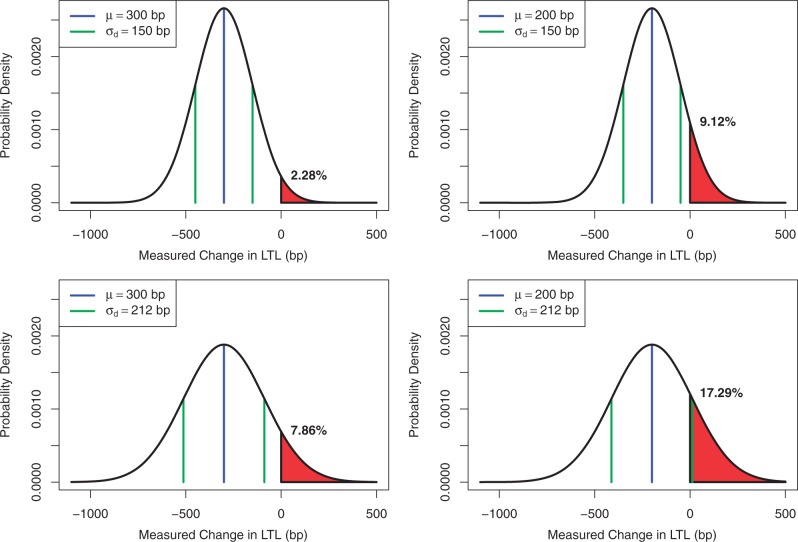
Theoretical curves showing the distribution of measured change in LTL because of measurement error. Red shaded areas denote the percentage of individuals who will be misclassified as LTL gainers. Left panels assume a loss of 300 bp and right panels assume a loss of 200 bp during follow-up periods, which could correspond to 10 years of follow-up with an attrition of either 30 or 20 bp/year, respectively. Top panels assume an SD of the measured difference of 150 bp, and bottom panels assume an SD of the measured difference of 212 bp, which could correspond to an SD of the measurement error of 150 bp for two independent measurements or just a single measurement, respectively. As we consider the measured change in LTL to be positive for LTL gainers, μ (200 or 300 bp) is positioned to the left of 0 on the *x*-axis.

The two tails of the normal distribution extend to infinity. Thus, no matter how large μ is and how small σ is, there will always be a chance that the measured change is positive. That said, a larger μ and a smaller σ will result in fewer individuals who appear to show a longer LTL at follow-up (LTL gainers). This is illustrated for different normal distributions in Figure 1. For instance, one distribution assumes that the true loss μ is 300 bp (which could correspond to 10 years of follow-up at a loss of 30 bp/year), and that the σ_d_ is 150 bp (which could correspond to σ of 150 bp with two replicates). The red shaded areas correspond to the individuals who (purely because of measurement error) are observed to have a longer LTL at follow-up than at baseline, i.e. LTL gainers. The probability of such a misclassification in this case is 2.28%, which can be found using computer software or standard tables for the normal distribution.

Unfortunately, the σ of the measurement error is typically not reported in publications on LTL. Instead, the unitless CV of LTL (i.e. the SD divided by the mean of LTL) is reported. Therefore, when converting the CV (inter-assay or intra-assay) to σ to construct the theoretical curves depicting the relation between the percentage of LTL gainers and follow-up duration (Figure 3), we assumed a mean LTL of 7 kb (which is equivalent to the average LTL in young adults).

**Figure 2.**
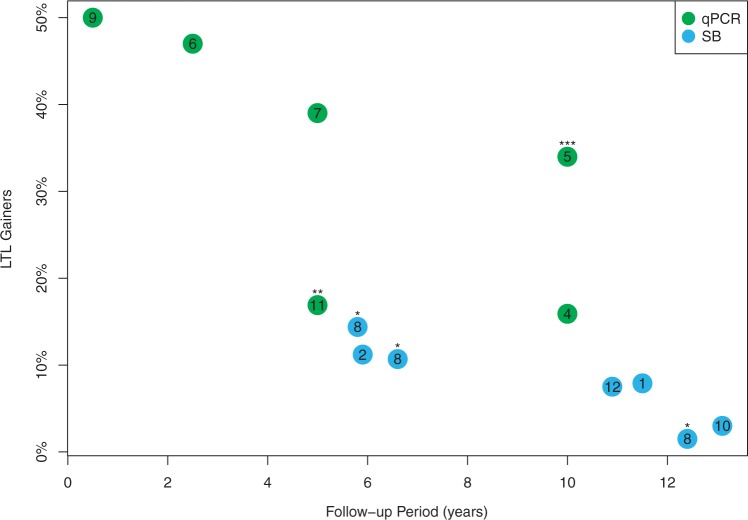
Percentage of LTL gainers versus follow-up time based on studies presented in Table 1. The number in each symbol denotes the reference of the study. *A subset of the same study; **this percentage of LTL gainers is an underestimate, as it does not include half the subgroup with ‘unchanged LTL’ (which was not provided by the authors); ***this percentage of LTL gainers is an overestimate, as it includes the entire ‘unchanged LTL’ subgroup.

Notably, Epel *et al.* (6) and Farzaneh-Far *et al.* (7) reported subsets of participants in their studies as ‘maintainers’ of LTL, i.e. unchanged LTL, based on a difference of follow-up LTL from baseline LTL of less than±15% and ±10%, respectively. Based on these definitions, 46% in Epel’s study (6) and 32% in Farzaneh-Far’s study (7) displayed unchanged LTL during the follow-up period. For estimating the actual percentage of LTL gainers, we assumed that half of these individuals, i.e. 23% in Epel’s study (6) and 16% in Frazaneh-Far’s study (7), were LTL gainers. This approach was applied to all studies listed in Table 1, even when the number of individuals reported as having unchanged LTL was small. Nordfjäll *et al.* (5) reported the percent of individuals with ‘stable or increased’ LTL; thus, the observed 34% of LTL gainers (Table 1 and Figure 2) might be an overestimate, based on the principle applied to the other studies. In contrast, Shalev *et al.* (11) reported only individuals showing >15% LTL lengthening as LTL gainers; thus, the observed 16.9% of LTL gainers (Table 1 and Figure 2) might be an underestimate, based on the same principle. In addition, we could not compute the predicted percentage of LTL gainers in a number of studies because of insufficient information, such as absent baseline LTL or follow-up LTL and lack of clarity about the CV. Specific reasons are also provided in the legend of Table 1.

## RESULTS

Based on empirical data derived from the longitudinal studies presented in Table 1, the proportion of LTL gainers was inversely related to the duration of the follow-up, i.e. the shorter the duration, the greater the proportion of observed LTL gainers (Figure 2). This was evident both for Southern blot (SB) analysis and quantitative polymerase chain reaction (qPCR)-based methods of LTL measurement. However, smaller proportions of LTL gainers were consistently observed for the SB analysis (Figure 2). It could be argued that although in the long run LTL undergoes age-dependent shortening in all individuals, it experiences true fluctuations up (being lengthened) and down (being shortened) during short time intervals, as previously suggested (9). To address this possibility, we performed computations of the impact of the measurement error on the outcome of longitudinal studies of LTL attrition.

**Figure 3.**
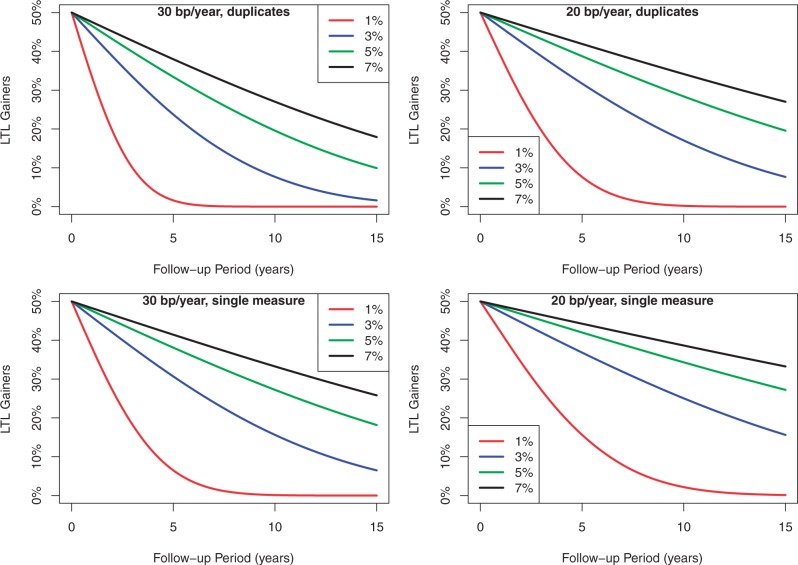
Theoretical curves showing the corresponding percentage of LTL gainers for a given follow-up period for different inter-assay CVs. Upper panels are based on two independent LTL measurements at baseline and two independent measurements at follow-up, and lower panels on one measurement at baseline and follow-up. Left panels display the curves for an average rate of LTL attrition of 30 bp/year. Right panels display the curves for an average rate of LTL attrition of 20 bp/year. Keys for trajectories of different CVs are shown in the insets.

Figure 3 was constructed based on the stipulation that (i) LTL = 7 kb at baseline, (ii) average rates of age-dependent LTL attrition of 30 or 20 bp/year and (iii) single or duplicate measurements of baseline and of follow-up LTLs. The figure showcases the computed percentage of individuals misclassified as LTL gainers for different measurement errors and follow-up periods. For instance, with a CV of 3%, ∼8% of individuals would be misclassified as LTL gainers when (i) duplicate measurements are performed at baseline and follow-up examinations, (ii) the rate of LTL attrition is 30 bp/year and (iii) the follow-up duration is 10 years. However, ∼20% of individuals would be misclassified as LTL gainers under the same circumstances when the CV is 5%. (Figure 3, left upper panel).

To further illustrate the impact of the measurement error of LTL on findings and conclusions, we focused on studies that provided sufficient information (i.e. the CV of the LTL measurement, the follow-up duration and the rate of LTL attrition based on baseline and follow-up LTL measurements) to compute the predicted percentage of LTL gainers (Table 1). The predicted proportion of LTL gainers ranged from 0.0 (4) to 50.0% (9), and the observed proportions of LTL gainers ranged from 3.0 (10) to 50% (9). In one study, the predicted and observed proportions of LTL gainers were the same, i.e. 50% (9). In another sub-study, the predicted percentage of LTL gainers was minimally larger (14.8%) than the observed value (14.4%) (8). In all other studies or sub-studies, the predicted proportions of LTL gainers were smaller than the observed values. However, in three of these studies, the predicted values (9.6, 1.0 and 1.8%) minimally differed from the observed ones (10.7, 1.5 and 3.0%, respectively). Thus, in five studies, the predicted percentages of those misclassified as LTL gainers matched well with the observed LTL gainers. In five other studies, the predicted percentages of LTL gainers (0.1, 1.0, 0.0, 1.7 and 22.8%), differed substantially from the, respectively, observed values (7.9, 11.2, 15.9, 7.5 and 39%).

## DISCUSSION

It is baffling that some authors did not consider TL measurement error as the most parsimonious explanation for their finding of LTL lengthening in longitudinal studies. Instead they concluded that LTL frequently elongates with age and sought a biological meaning for this phenomenon. Gardner *et al.* (1) were the first to report LTL lengthening in a small longitudinal study and postulated biological causes for this enigmatic finding. However, a recent report concluded that LTL lengthening observed in longitudinal studies is essentially an artifact, which is largely because of laboratory error in TL measurements in relation to the follow-up duration (8). Previously, the underlying reasons for this discrepancy were examined through empirical evaluation (8) and here through computations based on published empirical data and theoretical considerations.

The tenet that age-dependent LTL attrition might be halted during the human life course was originally proposed based on cross-sectional studies, which apparently showed that LTL attrition levels off during early adulthood (15,16). However, this claim was not persuasive, as has been made clear by statistical considerations (17). With necessarily guarded inference because of the limitations of the cross-sectional design, statistical considerations indicated that because of the wide inter-individual variation in LTL at a given age, large sample sizes and wide age ranges of the subjects are required in cross-sectional studies for a reliable assessment of age-dependent LTL attrition. Although the rate of LTL shortening is more rapid during early life than during adult life (15,18,19), based on numerous cross-sectional studies, including a large study recently published (19), there is no evidence for a pause in LTL shortening during early adulthood.

As we show here, longitudinal evaluations are also subject to factors that strongly affect estimates of age-dependent LTL attrition, namely, laboratory error, number of replicates, the length of follow-up and the rate of age-dependent LTL attrition. The duration of the follow-up period in relation to the CV of TL measurements is critical as demonstrated in Figures 1–3, given that the magnitude of change in LTL is usually but a fraction of the absolute value of LTL (20).

Three major factors might account for the lower predicted estimate of individuals misclassified as LTL gainers. First, investigators might underestimate their measurement error of LTL. The study by Farzaneh-Far *et al.* (Table 1) (7), which showed the largest gap between the observed (39% LTL gainers) versus the predicted (22.8% of individuals misclassified as such), could serve as an illustration. The predicted value of LTL gainers in that study was based on its reported inter-assay CV of 3.7% for LTL measurements by qPCR, but a recent impartial evaluation of the inter-assay CV of LTL in the laboratory performing these measurements was 6.4% (21) (the SB method was also evaluated impartially in that study, but, as shown in Table 1, its inter-assay CV of 1.7% was similar to values reported in longitudinal studies using this method). Computation based on a CV of 6.4% for the study by Farzaneh-Far *et al.* (7) yielded a predicted value of 33.4% of individuals being misclassified as LTL gainers, which is much closer to the observed value of LTL gainers reported by the authors. Notably, the effect of measurement error can be reduced in longitudinal studies in which the individual’s baseline and follow-up samples are assayed in the same ‘batch’ (qPCR) or ‘gel’ (SB). For further details, see Supplementary Supplement S1.

Second, the predicted percentage of LTL gainers is based on the assumption that LTL attrition is the same for all individuals, which, as noted under the ‘Materials and Methods’ section, is unlikely to be the case. A more realistic model to predict the percentage of LTL gainers would be a random effects model that accounts for between-individual variation in LTL longitudinal change. However, published papers rarely provide the entire data set that is necessary to apply the random effects model, which typically generates a higher predicted percentage of LTL gainers. The recently published study (12), whose entire data were available to us, serves to illustrate this concept. Using the model we use here, i.e. LTL attrition is the same in all individuals, the predicted percentage of LTL gainers was 1.7 (Table 1). However, using the random effects model, which includes the mean LTL and the individual’s specific attrition, the predicted percentage of LTL gainers was 10.6. This was much closer to the observed 7.5% (95% confidence interval 2.8–15.6) of LTL gainers reported in that study (12). For further details see Supplementary Supplement S2.

Third, although laboratory measurement error is a major cause, by no means is it the only factor that accounts for the misclassification of subjects. The circumstances related to the baseline and follow-up samples are also important. In longitudinal studies, blood samples are often collected and DNA extracted by different investigators and different techniques that might impact on DNA integrity and purity. Although the state of DNA integrity might affect LTL results generated by SB to a greater extent than those generated by qPCR, protein and organic solvent impurities in the DNA samples might exert a greater impact on the qPCR results than those of the SB. Thus, meticulous attention to these potential problems should be exercised in longitudinal studies.

In summary, one cannot totally exclude unusual circumstances where an individual might display lengthening of LTL because of biological causes. However, as our analysis shows, under most circumstances, findings of age-dependent LTL lengthening seem to be the outcome of a less than optimal study design and LTL measurement error that is disproportionally large in relation to the extent of LTL attrition during the follow-up period. Teasing apart the two factors that fashion LTL during the human life course, i.e. LTL at birth and its age-dependent shortening afterward, will provide a valuable and long-awaited insight into human telomere biology and its role in human aging. But this cannot be accomplished through longitudinal studies in which the noise of TL determination because of measurement error exceeds age-dependent change in LTL during inadequate durations of follow-up. The graphs we present, and the specific illustrations we provide might serve as useful guidelines for researchers in estimating the duration of the follow-up period required in relation to the CV of TL measurement. These graphs also showcase the fact that in most cases, LTL lengthening is not a biologically determined result; it is a mathematical artifact related to the measurement error.

Finally, there is a need for impartial large-scale evaluations of the suitability of current TL measurement methods—SB, qPCR, fluorescence *in situ* hybridization and single telomere length analysis (STELA)—for clinical/epidemiological research. These evaluations, we suggest, can only take place if they are sponsored by national funding agencies, e.g. the National Institutes of Health in the USA and the Medical Research Council in the UK, through partnership with investigators in the telomere field. Such agencies are investing millions of dollars annually in telomere research, the outcome of a large portion of which might be dubious because of faulty methodology and experimental design. Without the oversight of such agencies, translation of the great strides made in basic telomere biology into useful insight into aging-related disease, including cardiovascular disease and cancer, may be compromised in the foreseeable future.

## SUPPLEMENTARY DATA

Supplementary Data are available at NAR Online: Supplementary Table 1, Supplementary Methods and Supplementary Reference [22].

## FUNDING

National Institutes of Health (NIH) [AG030678]; National Program for Research Infrastructure 2007 from the Danish Agency for Science Technology and Innovation; Israel Science Foundation (ISF); US-Israel Binational Science Foundation (BSF). Funding for open access charge: NIH [R01AG030678A].

*Conflict of interest statement.* None declared.

## Supplementary Material

Supplementary Data
