# Supplementary material for: The telomere lengthening conundrum—artifact or biology?
Source: Nucleic Acids Res. 2013 May 11;41(13):e131. doi: 10.1093/nar/gkt370 (PMC3905906; doi:10.1093/nar/gkt370)
Supplement: Supplementary Data [file supp_41_13_e131__index.html]

The telomere lengthening conundrum—artifact or biology? — The telomere lengthening conundrum—artifact or biology? — Supplementary Data 

# The telomere lengthening conundrum—artifact or biology?

## Supplementary Data

files

**Files in this Data Supplement:**

- Supplementary Data - docx file
