# Supplementary material for: The telomere lengthening conundrum—artifact or biology?
Source: Nucleic Acids Res. 2013 May 11;41(13):e131. doi: 10.1093/nar/gkt370 (PMC3905906; doi:10.1093/nar/gkt370)
Supplement: Supplementary Data [file supp_gkt370_nar-00423-met-n-2013-File005.docx]

**Supplement**

**I.** The inter-assay error comes from the inherent differences between gels/batches and is assumed to be constant for any given gel/batch. We assume that the inter-assay error is normally distributed with mean zero and standard deviation (SD) σ_inter_. The intra-assay error comes from the inherent measurement error between replicate measures on the same gel/batch. We assume that the intra-assay error is normally distributed with mean zero and SD σ_intra_.

Inter-assay and intra-assay SDs can both be estimated using the equation:

[∑_i_(n_i_ - 1)s_i_^2^] / [∑_i_(n_i_ - 1)]. (1)

If there are n_i_ replicates of a sample on the same gel/batch, and s_i_ is the estimated SD of these n_i_ replicates, then σ_intra_^2^ is given by the above equation. If there are n_i_ replicates of a sample on different gels/batches, and s_i_ is the estimated SD of these n_i_ replicates, then σ_intra_^2^ + σ_inter_^2^ is given by the above equation.

The predicted % of LTL gainers is given by:

(1 - Φ(μ/σ_d_))*100%, (2)

where μ is the average attrition, Φ is the cumulative distribution function of the standard normal distribution and σ_d_ depends on the intra-assay deviation (σ_intra_), the inter-assay deviation (σ_inter_) and the specific setting for the arrangement of samples on the gels/batches (Supplementary Table 1). Settings (i) and (ii) are likely to provide more reliable results for the longitudinal evaluation of LTL attrition. We note that studies often fail to report both the intra-assay and the inter-assay CV. They also often do not provide details about the specific setting for their measurements of baseline and follow-up samples.

For simplicity, and since settings and reported CVs are rarely completely specified in publications, we have chosen to base the estimates on the more commonly reported inter-assay CV. This does not affect the analysis shown in any of the figures, but may influence the predicted % of LTL gainers in Table 1 and may be a further explanation for the discrepancies between observed and predicted LTL gainers^.^

**Supplementary Table 1.** Forulas for σ_d_ for various gel/batch combinations with two baseline (b) measurements and two follow-up (fu) measurements

| **gel/batch combination** | **σ_d_** |
| --- | --- |
| (i) all on same gel/batch | σ_intra_ |
| (ii) b and fu run in pairs | σ_intra_ |
| (iii) all on different gels/batches | (σ_intra_^2^ + σ_inter_^2^)^1/2^ |
| (iv) b on one gel/batch, fu on another | (σ_intra_^2^ + 2σ_inter_^2^)^1/2^ |

The mathematics behind Table 1 are as follows:

Let b_1_ and b_2_ denote the two baseline measurements and let fu_1_ and fu_2_ denote the two follow-up measurements (in combination (ii), the labels must match the pairs, i.e., b_1_ and fu_1_ form pair 1 and b_2_ and fu_2_ form pair 2. (In the other combinations the choice of label does not matter). The baseline and follow-up values can be expressed as:

b_i_ = b + N(σ_inter_^2^) + N(σ_intra_^2^) (i = 1,2) (3)

and

fu_i_ = fu + N(σ_inter_^2^) + N(σ_intra_^2^) (i = 1,2), (4)

where b and fu are the true (unknown) values and N(σ^2^) represent a normally distributed value with mean zero and standard deviation σ. Depending on the gel/batch combination, the contribution from the inter-assay variation is the same for some or all of the measurements, while the contribution from the intra-assay variation is always different for all of the measurements.

(i) The inter-variation is exactly the same for all measurements and hence cancels out when subtracting them, i.e.,

b_1_ - fu_1_ = b - fu + N(2σ_intra_^2^) and b_2_ - fu_2_ = b - fu + N(2σ_intra_^2^). (5)

Therefore,

change = (b_1_ - fu_1_)/2 + (b_2_ - fu_2_)/2 = b - fu + N(σ_intra_^2^). (6)

(ii) The inter-variation is exactly the same for each baseline/follow-up pair and hence cancel out when subtracting them, i.e.,

b_1_ - fu_1_ = b - fu + N(2σ_intra_^2^) and b_2_ - fu_2_ = b - fu + N(2σ_intra_^2^). (7)

Therefore,

change = (b_1_ - fu_1_)/2 + (b_2_ - fu_2_)/2 = b - fu + N(σ_intra_^2^). (8)

(iii) The inter-variation is different for all measurements and hence the mean of the baseline- and follow-up measurements are given by:

(b_1_ + b_2_)/2 = b + N(σ_inter_^2^/2) + N(σ_intra_^2^/2)

and (fu_1_ + fu_2_)/2 = fu + N(σ_inter_^2^/2) + N(σ_intra_^2^/2). (9)

Therefore,

Change = (b_1_ + b_2_)/2 - (fu_1_ + fu_2_)/2 = b - fu + N(σ_inter_^2^ + σ_intra_^2^). (10)

(iv) The inter-variation is exactly the same for all baseline measurements (one value) and all follow-up measurements (another value) and hence the mean of the baseline- and follow-up measurements are given by:

(b_1_ + b_2_)/2 = b + N(σ_inter_^2^) + N(σ_intra_^2^/2) and (fu_1_ + fu_2_)/2 = fu + N(σ_inter_^2^) + N(σ_intra_^2^/2). (11)

Therefore,

Change = (b_1_ + b_2_)/2 - (fu_1_ + fu_2_)/2 = b - fu + N(2σ_inter_^2^ + σ_intra_^2^). (12)

We note that the "missing one-half" in the inter-variation term is caused by the inability to correct for the gel/batch effect when performing measurements on the same gel/batch (no matter how many times a baseline measurement is replicated on the same gel, it will never be able to reduce the error caused by the gel/batch effect).

**II.** Based on equation 2 above, it can be shown that the true **%** of LTL gainers by chance will be greater than the estimate computed by assuming that all individuals have the same attrition. In other words, the **%** of LTL gainers obtained in this paper is conservative. The proof follows from Jensen's inequality (22), since equation 2 is a convex function of μ, as long as μ is positive (corresponding to LTL attrition).

Supplementary Reference

22. Tristan Needham (1993) A Visual Explanation of Jensen's Inequality. American Mathematical Monthly **100**(8):768-771.
